# Visual acuity is correlated with ischemia and neurodegeneration in patients with early stages of diabetic retinopathy

**DOI:** 10.1186/s40662-021-00260-4

**Published:** 2021-10-19

**Authors:** Jin Li, Yue Zhou, Feng Chen, Yingzi Li, Rong Zhou, Chaoming Wu, Huankai Yu, Zhiyang Lin, Ce Shi, Gu Zheng, Yilei Shao, Qi Chen, Fan Lu, Meixiao Shen

**Affiliations:** 1 School of Ophthalmology and Optometry, Wenzhou Medical University, 270 Xueyuan Road, Wenzhou, 325027 Zhejiang China; 2 The Second Affiliated Hospital and Yuying Children’s Hospital of Wenzhou Medical University, Wenzhou, 325027 Zhejiang China; 3 Department of Ophthalmology, The Affiliated Hospital of Yangzhou University, Yangzhou, 225000 Jiangsu China

**Keywords:** Diabetic retinopathy, Visual acuity, Optical coherence tomography, Ischemia, Neurodegeneration

## Abstract

**Purpose:**

We investigated the effects of retinal ischemia, neurodegeneration, and subclinical edema on best-corrected visual acuity (BCVA) in the early stages of diabetic retinopathy (DR).

**Methods:**

Ischemia was evaluated by the microvascular parameters measured by optical coherence tomography angiography. Neurodegeneration and subclinical edema were identified by the intraretinal layer thickness obtained by optical coherence tomography. Eyes with nonproliferative diabetic retinopathy (n = 132) from 89 patients were analyzed. Eyes were classified as having normal BCVA (n = 88 [66.7%], Snellen equivalent ≥ 20/20) or decreased BCVA (n = 44 [33.3%], Snellen equivalent < 20/20). The prevalence of ischemia, neurodegeneration, and subclinical edema was explored in patients with and without decreased BCVA, and correlations between BCVA and these pathological pathways were determined.

**Results:**

Vessel density in the deep retinal capillary plexus (DRCP) and thickness of ganglion cell layer plus inner plexiform layer (GCL-IPL) were significantly lower in eyes with decreased BCVA compared with eyes with normal BCVA (both *P* < 0.05). In the final multiple regression predictive model, age, DRCP vessel density, and GCL-IPL thickness (all *P* ≤ 0.044) were predictors of BCVA. DRCP vessel density and GCL-IPL thickness have an interactive effect on visual acuity. The proportions of ischemia and neurodegeneration were significantly higher in eyes with decreased BCVA than in eyes with normal BCVA (*P* = 0.001 and *P* = 0.004, respectively).

**Conclusion:**

During the natural course of the early stages of DR, ischemia and neurodegeneration were the main disease pathways associated with visual acuity, and the mechanisms varied among patients.

**Supplementary Information:**

The online version contains supplementary material available at 10.1186/s40662-021-00260-4.

## Background

Diabetic retinopathy (DR) is a leading cause of vision impairment and blindness among working adults worldwide [1]. In recent years, DR has been characterized by the predominance of one of the three phenotypes: ischemia, neurodegeneration, and edema [2–4]. These three phenotypes vary greatly among different diabetic patients and lead to different risks for development of vision-threatening complications [5]. In view of the relevance of DR as a public health issue and a huge financial burden to government agencies, insurance companies, and individuals [6], it is important to improve our understanding of the mechanisms of the three phenotypes and the roles they play in the vision loss that occurs during the initial stages of DR. This information may help to develop improved early and individualized intervention strategies.

DR is conventionally described as a microvasculopathy; thus, the gold standard of severity classification is also based on this concept [7], and greater levels of DR severity usually correspond to worse vision loss. However, this correlation is very weak in nonproliferative diabetic retinopathy (NPDR) [8, 9]. This indicates that microvascular changes are not the only pathological factor of the early visual impairment, and other mechanisms or metabolic changes are also implicated. In addition, many studies have indicated that ischemic and microvascular changes may lead to neurodegeneration or macular edema [10]. It is unclear if there are any correlations of the ischemic, neurodegenerative, and edematous phenotypes with vision loss. Consequently, there is no information regarding any potential interaction of these phenotypes that can affect visual acuity in the early stage of DR in patients who have no apparent macular complications. This is because commonly measured parameters, such as the size of the foveal avascular zone (FAZ) assessed by fluorescein angiography and whole retinal thickness assessed by optical coherence tomography (OCT) are of limited use for characterizing ischemia, neurodegeneration, and edema present in the early stages of DR.

Recently, investigators have used OCT with a sublayer segmentation algorithm to quantify the neurodegeneration characterized by the thinning of retinal nerve fiber layer (RNFL) and ganglion cell layer plus inner plexiform layer (GCL-IPL) [11–13]. OCT can also detect subclinical edema that is not observed clinically. It is diagnosed based on the thickness of the inner nuclear layer (INL), outer plexiform layer (OPL), and total retina that exceed the normal values [14]. In addition, ischemia can be identified by decreased vessel density and increased FAZ using optical coherence tomography angiography (OCTA) [15–17]. Therefore, OCT and OCTA offer the possibility of quantitatively assessing the associations among the three phenotypes with the loss of visual acuity in the initial stages of DR. The goal of our study was to use OCT and OCTA to quantify three pathological alterations, i.e., ischemia, neurodegeneration, and subclinical edema in the early stages of type 2 DR and to explore the correlations with visual acuity.

## Methods

### Subject data collection

Of the 89 patients with type 2 diabetes mellitus (DM) who participated in this study, 69 were enrolled from the Endocrinology Department of the Second Affiliated Hospital of Wenzhou Medical University, Wenzhou, China, where they were diagnosed by an endocrine specialist (CW). Twenty patients were enrolled from the Fundus Department at the Eye Hospital of Wenzhou Medical University. To serve as a control reference group for comparison with the study group of DR patients, an additional 33 healthy subjects (21 women and 12 men; 56.9 ± 7.3 years) were recruited from workers at the Eye Hospital of Wenzhou Medical University and family members of patients at the same hospital. With the exception of having DR, the inclusion and exclusion criteria were the same as for the DR patients. This study followed the tenets of the Declaration of Helsinki and was approved by the ethics committee of the Eye Hospital of Wenzhou Medical University. Written informed consent was obtained from all patients.

All subjects underwent a series of ophthalmologic examinations, including slit-lamp biomicroscopy, intraocular pressure (IOP) measurement and ophthalmoscopy. After a full objective and subjective refraction, the best-corrected distance visual acuity was determined monocularly in logarithm of the minimum angle of resolution (logMAR). The DR severity level was determined independently by two experienced graders and was based on the 7-field protocol using the ETDRS classification. Only patients with type 2 DM and NPDR were enrolled. Potential subjects and controls were excluded if they had lens or other ocular media opacities preventing detailed imaging, high myopia (under − 6.00 diopters), clinical evidence of any other maculopathy, glaucoma or other neurodegenerative conditions, or any previous treatment of DR. Patients with a current or previous history of diabetic macular edema, defined as a central subfield thickening of at least 275 μm on spectral domain OCT (SD-OCT) [18], were also excluded. Demographic information collected from the patients included age, sex, body mass index, mean arterial pressure, duration of DM, and fasting blood glucose.

Based on best-corrected visual acuity (BCVA) levels, eyes of the diabetic cohort were divided into two groups: eyes with normal BCVA (logMAR ≤ 0 and Snellen equivalent ≥ 20/20) and eyes with decreased BCVA (logMAR > 0 and Snellen equivalent < 20/20). Eyes were also classified into three groups according to the ETDRS level: no or minimal NPDR, level 20; mild NPDR, level 35; moderate or severe NPDR, level 43–53.

### OCTA and quantitative analysis of the macular capillary plexuses

After pupillary dilation, the retinal microvasculature was imaged in a dark room by the RTVue XR Avanti SD-OCT system (Optovue, Inc., Fremont, CA, USA) equipped with AngioVue software (Version 2017.1.0.155). We chose the macular 3 × 3-mm program, with a scan speed at 70,000 A-scans per second and pixel dimensions of 304 × 304. OCTA images with significant image artifacts and poor image quality (signal strength index < 40) were excluded, an example is given in Additional file 1: Fig. S1. The superficial retinal capillary plexus (SRCP) and deep retinal capillary plexus (DRCP) were detected and separated automatically.

**Fig. 1:**
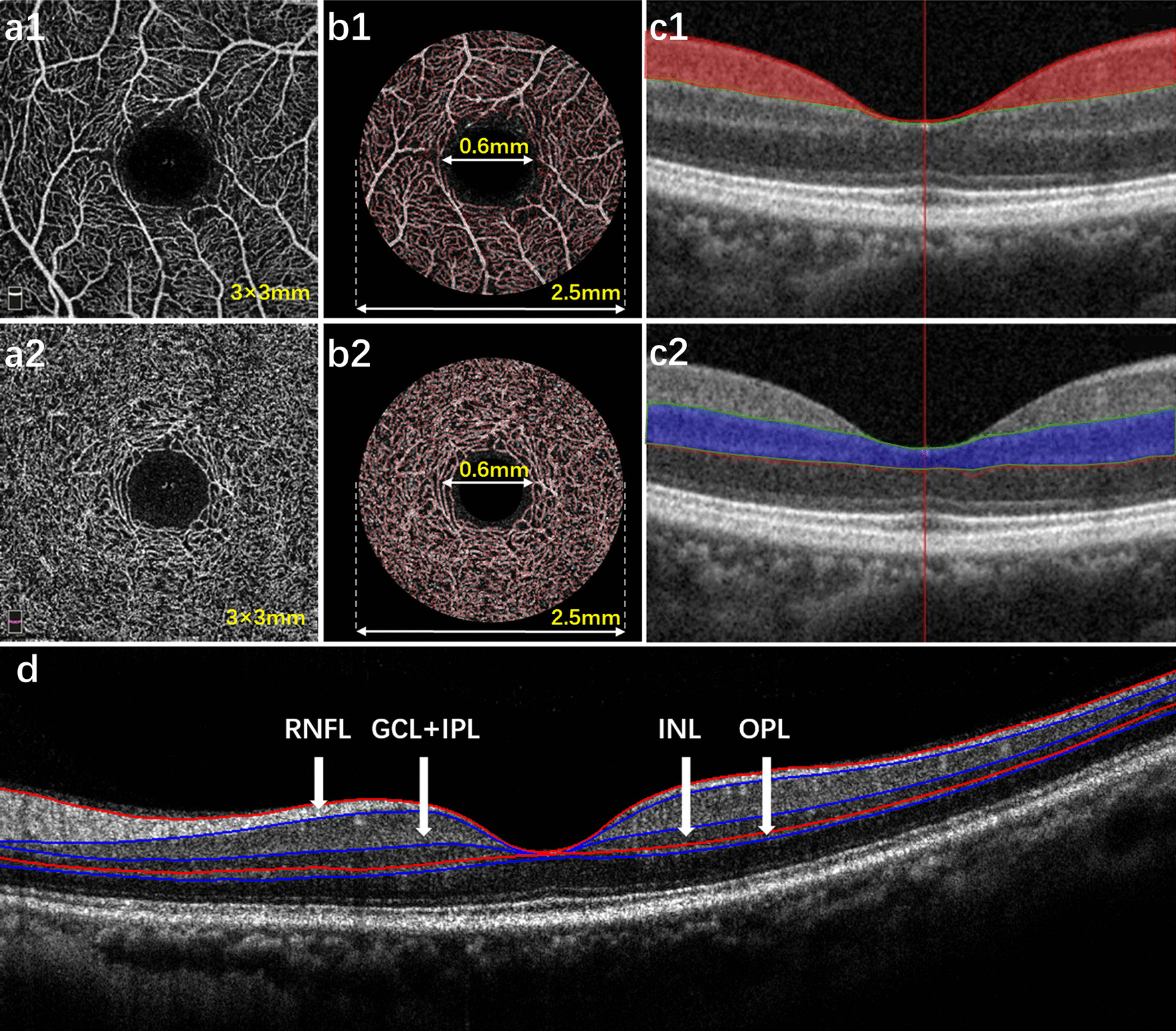
Optical coherence tomography angiography (OCTA) images showing vessel density analysis of the superficial and deep retinal capillary plexuses (SRCP and DRCP) and the intraretinal layers analyzed by custom software. OCTA images of the SRCP (**a1**) and DRCP (**a2**) in the 3 × 3-mm area around the fovea. Binary images of the microvascular network in the SRCP (**b1**) and DRCP (**b2**) showing the density in the annular zone with a diameter of 2.5 mm after excluding the foveal avascular zone (diameter = 0.6 mm). **c1** The SRCP (red) is shown as a slab extending from the internal limiting membrane to 9 μm above inner plexiform layer (IPL). **c2** The DRCP (blue) is shown as a slab extending from 9 μm above IPL to 9 μm below outer plexiform layer. **d** Intraretinal layer structures in horizontal scan OCT images: RNFL, retinal nerve fiber layer; GCL-IPL, ganglion cell layer plus inner plexiform layer; INL, inner nuclear layer; OPL, outer plexiform layer

A custom automated algorithm was used to quantify the SRCP and DRCP vessel densities in the *en face* OCTA projection images as described previously [13, 19, 20]. In brief, images were exported from the OCTA device. To enhance the details, the grayscale of each two-dimensional OCTA image was first extended by bicubic interpolation to 1024 × 1024 pixels. Then, the image was segmented to obtain the microvascular networks. The vessel density was calculated for the 2.5-mm diameter annular zone after excluding the FAZ (diameter = 0.6 mm, Fig. 1a, b). The methods above were implemented using MATLAB v. 7.10 (MathWorks, Inv., Natick, MA, USA). The SRCP was defined as a slab extending from the internal limiting membrane to 9 μm above the inner plexiform layer (IPL) (Fig. 1c1). The DRCP was defined as a slab extending from 9 μm above the IPL to 9 μm below the OPL (Fig. 1c2). The FAZ area was calculated using the area function on the integrated automated algorithms 2.0 software.

### SD-OCT measurement of individual retinal layer thicknesses

As described previously [11], each cross-sectional image from the “radial” mode of the OCT scans was analyzed by one masked reader (JL) to segment the boundaries of the intraretinal layers, and the average thickness was calculated. Automatic segmentation was achieved by a custom software program based on the gradient information and shortest path search that was developed in MATLAB for image analysis (Fig. 1d). Each image was manually confirmed through visual inspection by a masked grader after automatic segmentation. The central retinal thickness was defined as the average thickness (μm) in the central 1-mm circle defined by ETDRS. The central retinal thickness of the INL, OPL, and total retina were used to evaluate subclinical edema. Neurodegenerative changes were estimated based on RNFL and GCL-IPL average thicknesses in a central 6-mm circle.

### Statistical analysis

Data analyses were performed using the SPSS software (SPSS v.22.0, Chicago, IL). The t-test, ANOVA or Kruskal–Wallis test (for continuous variables) and χ^2^ test (for categorical variables) were used to analyze differences between each cohort. The mean and standard deviation (SD) values of the healthy control group served as reference values. Changes were considered to be obvious if different from normal values by more than 1.96 SD. Univariate linear regression models were fit using age, DM duration, ETDRS level, eye, vessel density, FAZ area, and intraretinal or total layer thickness as a single predictor, with BCVA as the outcome. Results from univariate regression models were then used to create a multivariate model with BCVA as the outcome. Parameters that were statistically significant were used to construct the final multivariate regression model and evaluated for the presence of any interactions. To adjust the inter-eye correlation from the same participant, as some patients had bilateral imaging, and to consider possible different demographic characteristics, the generalized estimating equations (GEE) method was used throughout the analysis whenever applicable. A value of *P* less than 0.05 was considered statistically significant.

## Results

### Subject data collection

Eyes without DR (n = 25), with diabetic macular edema (n = 9), proliferative diabetic retinopathy (n = 1), or history of treatment (n = 3) were excluded. Among the 89 subjects with type 2 diabetes, 145 eyes were potentially eligible for this study. After quality checks, only 132 eyes were included in the final analysis. Of the 89 diabetic patients (132 eyes), 53 patients (88 eyes, 66.7%) had a normal BCVA with a mean (SD) BCVA of − 0.04 (0.06) logMAR. Thirty-six patients (44 eyes, 33.3%) had decreased BCVA with a BCVA of 0.12 (0.14) logMAR. Table 1 summarizes the demographic and ocular findings of the groups with normal and decreased BCVA.

**Table 1:** Control and diabetic retinopathy population and ocular characteristics

	Control	DR	DR with normal BCVA (G1)	DR with decreased BCVA (G2)	*P* value		
					All groups	Control vs. DR	G1 vs. G2
Population characteristics (n = 89)							
No. of participants, n	33	89	53	36	NA	NA	NA
Age (years)	56.94 ± 7.32	54.66 ± 10.95	50.72 ± 9.43	60.47 ± 10.55	< 0.001^a^	0.960^a^	< 0.001^a^
Sex (M/F)	12/21	54/35	36/17	18/18	0.014^b^	0.017^b^	0.089^b^
BMI (kg/m^2^)	24.57 ± 2.49	25.40 ± 3.67	25.56 ± 3.35	25.16 ± 4.15	0.433^a^	0.452^a^	0.598^a^
MAP (mmHg)	92.42 ± 10.61	96.41 ± 22.61	96.78 ± 24.11	95.82 ± 20.29	0.079^c^	0.372^c^	1.000^c^
DM duration (years)	NA	9.06 ± 6.34	7.62 ± 5.27	11.19 ± 7.21	NA	NA	0.021^c^
BG (mmol/L)	NA	7.78 ± 2.64	7.42 ± 2.46	8.35 ± 2.83	NA	NA	0.067^a^
Ocular characteristics (n = 132)							
No. of eyes, n	33	132	88	44	NA	NA	NA
BCVA (logMAR)	− 0.05 ± 0.05	0.01 ± 0.12	− 0.05 ± 0.06	0.12 ± 0.14	< 0.001^c^	0.008^c^	< 0.001^c^
SE (D)	0.16 ± 1.16	− 0.10 ± 1.39	− 0.13 ± 1.42	0.21 ± 1.27	0.009^c^	0.071^c^	0.038^c^
IOP (mmHg)	12.53 ± 2.42	14.41 ± 3.48	14.20 ± 3.53	14.83 ± 3.37	0.012^a^	0.005^a^	0.306^a^
DR severity							
ETDRS 20 (n = 56)	NA	56	39	17	NA	NA	0.534^b^
ETDRS 35 (n = 52)	NA	52	33	19	NA	NA	0.529^b^
ETDRS 43–53 (n = 24)	NA	24	16	8	NA	NA	1.000^b^

*BCVA* best-corrected visual acuity; *NA* not applicable; *M/F* male/female; *BMI* body mass index; *MAP* mean arterial pressure; *DM* diabetes mellitus; *BG* blood glucose; *logMAR* logarithm of the minimum angle of resolution; *IOP* intraocular pressure; *DR* diabetic retinopathy; *ETDRS* Early Treatment Diabetic Retinopathy

^a^t-test or ANOVA

^b^χ^2^ test

^c^Kruskal-Wallis test

### Different disease pathways in diabetic retinopathy

#### Ischemia

**Fig. 2:**
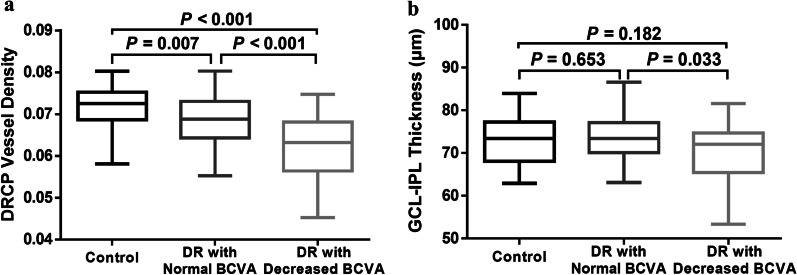
Comparison of deep retinal capillary plexus (DRCP) vessel density and ganglion cell layer plus inner plexiform layer (GCL-IPL) thickness between control group, diabetic retinopathy (DR) eyes with normal best-corrected visual acuity (BCVA) and DR eyes with decreased BCVA. The vessel density in DRCP (**a**) and thickness of GCL-IPL (**b**) are significantly lower in eyes with DR and decreased BCVA than in eyes with DR and normal BCVA

Ischemia, represented by decreased vessel density and enlargement of the FAZ, was present in 19.3% of DR eyes with normal BCVA and 47.7% of DR eyes with decreased BCVA (*P* = 0.001, Table 2). It was also present in 14.3%, 25.0%, and 70.8% of the eyes in ETDRS groups level 20, 35, and 43–53, respectively (*P* < 0.001, Table 2). After adjusting for confounding factors of age and DM duration, there were significant differences in vessel density in SRCP, DRCP, and the FAZ area among the three groups (Table 3). The DRCP vessel density was decreased in DR patients with decreased BCVA compared to DR patients with normal BCVA (0.062 vs. 0.069, *P* < 0.001, Table 3, Fig. 2a). Neither the vessel density in the SRCP nor the FAZ area differed significantly between patients with and without decreased BCVA changes (Table 3).

**Table 2:** Number of patients with ischemia, neurodegeneration, and subclinical edema by BCVA and ETDRS severity groups

	Normal BCVA (n = 88)		Decreased BCVA (n = 44)		*P* value	ETDRS 20 (n = 56)		ETDRS 35 (n = 52)		ETDRS 43–53 (n = 24)		*P* value
	No	% (95% CI)	No	% (95% CI)		No	% (95% CI)	No	% (95% CI)	No	% (95% CI)	
Ischemia												
VD SRCP decrease	11	12.5 (5.6–19.4)	10	22.7 (10.3–35.1)	0.130	4	7.1 (0.4–13.9)	10	19.2 (8.5–29.9)	7	29.2 (11.0–47.4)	**0.031**
VD DRCP decrease	12	13.6 (6.5–20.8)	19	43.2 (28.5–57.8)	** < 0.001**	6	10.7 (2.6–18.8)	9	17.3 (7.0–27.6)	16	66.7 (47.8–85.5)	** < 0.001**
FAZ area increase	2	2.3 (0–5.4)	3	6.8 (0–14.3)	0.333	1	1.8 (0–5.3)	2	3.8 (0–9.1)	2	8.3(0–19.4)	0.333
VD decrease or FAZ area increase	17	19.3 (11.1–27.6)	21	47.7 (33.0–62.5)	**0.001**	8	14.3 (5.1–23.5)	13	25.0 (13.2–36.8)	17	70.8 (52.6–89.0)	** < 0.001**
Neurodegeneration												
RNFL thinning	0	0	2	4.5 (0–10.7)	0.109	1	1.8 (0–5.3)	0	0	1	4.2 (0–12.2)	0.485
GCL-IPL thinning	0	0	4	9.1 (0.6–17.6)	**0.011**	1	1.8 (0–5.3)	2	3.8 (0–9.1)	1	4.2 (0–12.2)	0.672
RNFL or GCL-IPL thinning	0	0	5	11.4 (2.0–20.7)	**0.004**	2	3.6 (0–8.4)	2	3.8 (0–9.1)	1	4.2 (0–12.2)	1.000
Subclinical edema												
INL thickening	3	3.4 (0–7.2)	2	4.5 (0–10.7)	1.000	2	3.6 (0–8.4)	0	0	3	12.5 (0–25.7)	**0.031**
OPL thickening	14	15.9 (8.3–23.6)	7	15.9 (5.1–26.7)	1.000	10	17.9 (7.8–27.9)	5	9.6 (1.6–17.6)	6	25 (7.7–42.3)	0.201
Total retinal thickening	0	0	0	0	NA	0	0	0	0	0	0	NA
INL or OPL or total retinal thickening	16	18.2 (10.1–26.2)	7	15.9 (5.1–26.7)	0.746	11	19.6 (9.2–30.0)	5	9.6 (1.6–17.6)	7	29.2 (11.0–47.4)	0.094

*BCVA* best-corrected visual acuity; *ETDRS* Early Treatment Diabetic Retinopathy Study; *CI* confidence interval; *NA* not applicable; *VD* vessel density; *SRCP* superficial retinal capillary plexus; *DRCP* deep retinal capillary plexus; *FAZ* foveal avascular zone; *RNFL* retinal nerve fiber layer; *GCL-IPL* ganglion cell layer plus inner plexiform layer; *INL* inner nuclear layer; *OPL* outer plexiform layer

Pathological changes in ischemia, neurodegeneration, and subclinical edema were accepted if the differences between the normal values, i.e., the mean value of healthy the control population and the DR group values were more than 1.96 standard deviation (SD) units. Thus, ischemia was diagnosed if the value of DR group was 1.96 SD less than the normal value of the control group. Neurodegeneration was diagnosed if the value of DR group was 1.96 SD less than that of the control group. Subclinical edema was diagnosed if the value of DR group was 1.96 SD more than normal value. *P*-values were based on the Chi-squared test result of the ratio of each group. Bold font indicates significance of *P* < 0.05

**Table 3:** Comparison of retinal microvascular parameters and retinal sublayer or total thickness in control and diabetic groups after adjusting for age, duration of DM

	Control (G0)	DR With normal BCVA (G1)	DR with decreased BCVA (G2)	*P* All groups	*P* G0 vs. G1	*P* G0 vs. G2	*P* G1 vs. G2
Microvascular parameters							
VD SRCP	0.056 ± 0.004	0.054 ± 0.005	0.052 ± 0.006	**0.004**	**0.038**	**0.002**	0.128
VD DRCP	0.072 ± 0.005	0.069 ± 0.006	0.062 ± 0.007	** < 0.001**	**0.007**	** < 0.001**	** < 0.001**
FAZ area (mm^2^)	0.325 ± 0.132	0.342 ± 0.117	0.401 ± 0.097	**0.019**	0.466	**0.008**	0.196
Retinal layer thickness							
RNFL thickness (μm)	28.46 ± 2.88	30.04 ± 3.14	29.04 ± 3.64	0.214	0.082	0.272	0.849
GCL-IPL thickness (μm)	72.40 ± 5.59	73.62 ± 4.82	70.24 ± 6.21	0.112	0.653	0.182	**0.033**
INL thickness (μm)	17.52 ± 3.66	17.10 ± 3.41	17.10 ± 3.82	0.852	0.706	0.572	0.943
OPL thickness (μm)	10.15 ± 1.49	10.89 ± 2.51	10.60 ± 2.36	0.240	0.108	0.217	0.666
Total retinal thickness (μm)	239.46 ± 21.01	240.69 ± 16.13	236.31 ± 19.14	0.663	0.866	0.538	0.680

*DM*  diabetes mellitus; *BCVA* best-corrected visual acuity; *SRCP* superficial retinal capillary plexus; *DRCP* deep retinal capillary plexus; *VD* vessel density; *FAZ* foveal avascular zone; *RNFL* retinal nerve fiber layer; *GCL-IPL* ganglion cell layer plus inner plexiform layer; *INL* inner nuclear layer; *OPL* outer plexiform layer

Bold font indicates significance of *P* < 0.05

### Neurodegeneration

For DR eyes with normal BCVA, there was no detectable thinning of the RNFL or GCL-IPL (Table 2). In contrast, for eyes with decreased BCVA, 11.4% had neurodegenerative changes (*P* = 0.004 vs. eyes with normal BCVA). Neurodegeneration, represented by thinning of the RNFL and GCL-IPL, was present in 3.6%, 3.8%, 4.2% of the eyes in ETDRS groups level 20, 35, and 43–53, respectively (*P* = 1.000, Table 2). After adjusting for age and the duration of DM, there were no detectable differences in the thickness of the RNFL among the control, DR with normal BCVA, and DR with decreased BCVA groups (*P* > 0.05, Table 3). The thickness of the GCL-IPL in eyes with normal BCVA, 73.62 µm, was greater than in eyes with decreased BCVA, 70.24 µm (*P* = 0.033, Table 3, Fig. 2b). Therefore, we found that most of the neurodegenerative changes took place in the GCL-IPL.

### Subclinical edema

Subclinical edema, indicated by increased retinal thickness, was present in 18.2% of the DR eyes with normal BCVA and 15.9% of the DR eyes with decreased BCVA (*P* = 0.746, Table 2). It was present in 19.6%, 9.6%, and 29.2% of the eyes in ETDRS groups level 20, 35, and 43–53, respectively (*P* = 0.094, Table 2). After adjusting for age and duration of DM, the thicknesses of the INL, OPL, and total retina were similar among the three groups (*P* > 0.05 for all, Table 3).

### Overall distribution

**Fig. 3:**
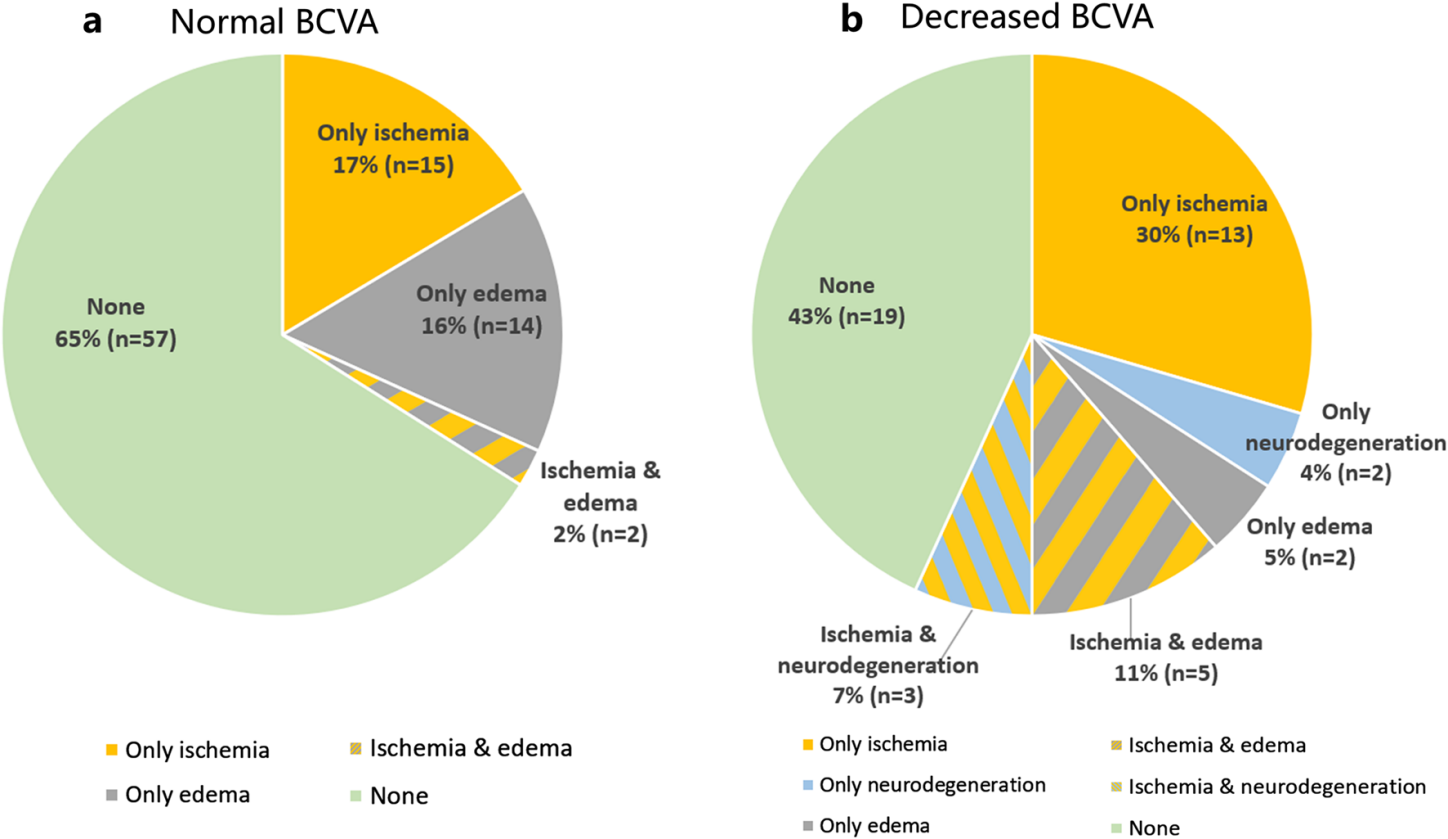
Distribution of eyes with different pathological conditions in diabetic retinopathy (DR) with normal best-corrected visual acuity (BCVA) and DR with decreased BCVA groups. Eyes with only one lesion appear as one color, and two lesions appear as two mixed colors

In DR eyes with normal BCVA, 65% did not have any obvious alteration in either ischemia, neurodegeneration, or subclinical edema. Among the DR subjects with normal BCVA eyes, 17% and 16% showed a single pattern of ischemia and subclinical edema, respectively, and 2% had both pathological manifestations. However, in DR eyes with decreased BCVA, only 43% had no detectable vascular or neurodegenerative changes (Fig. 3). Among the eyes with detectable changes, 39% had a single pathological mechanism, of which 30% had ischemia, 4% had neurodegeneration, and 5% had subclinical edema. Interestingly, some patients had two different pathological indicators. Thus, 11% of the eyes with reduced BCVA had both ischemia and subclinical edema, and 7% had ischemic and neurodegeneration simultaneously.

### Associations of different disease pathways with visual acuity in DR

Univariate GEE analyses showed that age (*P* < 0.001), duration of DM (*P* = 0.011), DRCP vessel density (*P* = 0.002), FAZ area (*P* = 0.022), and GCL-IPL thickness (*P* < 0.001) were significantly associated with BCVA. ETDRS level, eye, SRCP vessel density, and thickness of the RNFL, INL, OPL, and total retinal were not associated with BCVA (all *P* ≥ 0.05, Table 4). The significant predictors of BCVA in the univariate GEE analyses were then used in the final predictive model. Age, DRCP vessel density, and GCL-IPL thickness remained as significant predictors of BCVA in the final model (Table 4). Analysis of the receiver operating characteristics was used to calculate the cutoff value that determined the presence of visual impairment, defined as BCVA worse than 20/20 (Additional file 2: Table S1). Based on multiple regression models that affected the BCVA outcome, we explored the interactions between the GCL-IPL thickness and DRCP vessel density with BCVA as the outcome (Table 5), and the interaction was observed to be significant (*P* < 0.001).

**Table 4:** Results of simple and multiple regression models based on best corrected visual acuity outcome

Parameters	Simple regression model				Multiple regression model			
	Coefficient	Standard error	95% CI	*P* value	Coefficient	Standard error	95% CI	*P* value
Age	0.003	0.001	0.001 to 0.005	** < 0.001**	0.002	0.001	0.001 to 0.004	**0.009**
DM duration	0.004	0.002	0.001 to 0.007	**0.011**	–	–	–	–
ETDRS level	0.002	0.001	− 0.0003 to 0.004	0.087	–	–	–	–
Eye	0.007	0.017	− 0.027 to 0.041	0.686	–	–	–	–
VD SRCP	− 3.906	1.993	− 7.813 to 0.001	0.050	–	–	–	–
VD DRCP	− 7.258	2.344	− 11.852 to − 2.663	**0.002**	− 6.194	2.605	− 11.298 to − 1.089	**0.017**
FAZ area	0.448	0.195	0.065 to 0.830	**0.022**	–	–	–	–
RNFL thickness	− 0.005	0.003	− 0.012 to 0.001	0.120	–	–	–	–
GCL-IPL thickness	− 0.007	0.002	− 0.010 to − 0.004	** < 0.001**	− 0.004	0.002	− 0.007 to − 0.001	**0.044**
INL thickness	− 0.0005	0.003	− 0.006 to 0.005	0.856	–	–	–	–
OPL thickness	− 0.001	0.005	− 0.010 to 0.009	0.912	–	–	–	–
Total thickness	− 0.002	0.001	− 0.004 to 0.0002	0.079	–	–	–	–
Constant	NA	NA	NA	NA	0.575	0.196	0.191 to 0.959	**0.003**

*CI* confidence interval; *DM* diabetes mellitus; *ETDRS* Early Treatment Diabetic Retinopathy Study; *VD* vessel density; *SRCP* superficial retinal capillary plexus; *DRCP* deep retinal capillary plexus; *FAZ* foveal avascular zone; *RNFL* retinal nerve fiber layer; *GCL-IPL* ganglion cell layer plus inner plexiform layer; *INL* inner nuclear layer; *OPL* outer plexiform layer; *NA* not applicable

Bold font indicates significance of *P* < 0.05

**Table 5:** Multiple regression models for interactions between the GCL-IPL thickness and deep retinal capillary plexus vessel density based on best corrected visual acuity outcome

Parameters	Coefficient	Standard error	95% CI	*P* value
GCL-IPL thickness × VD DRCP	− 0.083	0.020	− 0.121 to − 0.144	< 0.001

*GCL-IPL* ganglion cell layer plus inner plexiform layer; *VD* vessel density; *DRCP* deep retinal capillary plexus

## Discussion

We utilized OCT- and OCTA-derived anatomic and microvascular parameters to investigate the visual significance of three different pathological pathways, i.e., ischemia, neurodegeneration, and subclinical edema in the natural progression of the early stages of DR. We then determined if these factors mutually influenced vison loss. The major findings of this study are as follows: (1) the prevailing mechanism of visual acuity loss may be different in different patients at the initial stage of DR; (2) we found that both ischemia, evaluated by OCTA-documented loss of vessel density in the DRCP, and neurodegeneration, evaluated by OCT-documented thinning of the GCL-IPL, were independently correlated with decreases in BCVA; (3) ischemia and neurodegeneration mutually influenced each other in affecting vision loss. Therefore, preventing further neurodegeneration as well as ischemia should be an important clinical goal for applying precision medicine in early diabetic retinal disease.

Our findings may contribute to the individual management of DR in the context of preventing early threats to vision. In our study, some DR eyes were found to manifest a single phenotype, i.e., ischemia or neurodegenerative. Interestingly, a portion had overlapping ischemia and neurodegeneration or ischemia and subclinical edema. These changes suggest that the predominant mechanism of visual impairment may be different in different patients, and indicators based on OCT and OCTA may help identify factors that threaten a patient’s vision, thereby guiding us to optimize the individualized treatment methods. Further longitudinal studies are required to determine if a personalized assessment of these pathways would protect the BCVA during the development of DR.

In this study, we found that of the three different disease pathways, ischemia was the main factor that threatened vision in the early stages of DR, which is in concordance with the conclusions of other reports [21–24]. In addition to ischemia, we discovered that neurodegeneration also plays a huge role in the loss of BCVA even in the early stages of DR. Further, we found that the thickness of the GCL-IPL was a more sensitive biomarker of early DR visual changes than RNFL. Reductions in the neurological layers are likely to indicate a reduced abundance of retinal ganglion cell axons, and probably a loss of cell bodies and dendrites. This defect may become an obstacle to the transmission of visual information to the brain and damage the information processing capabilities of the inner retina. Several studies also showed the early neurodegeneration in diabetic patients [25–27]. Alteration in retinal trophic factors, oxidative stress, and mitochondrial damage induced by hyperglycemia, low-grade inflammation, immune cell activation, and extracellular glutamate accumulation are crucial for the development of retinal neurodegeneration [28, 29]. However, the decrease of GCL-IPL thickness was not so evident and the casual relationships between neurodegeneration and BCVA decline was unclear in the current study. We assume that neurodegeneration may be more related to other visual function defects such as electroretinography, microperimetry, contrast sensitivity, and color vision, all of which could be evaluated in future studies.

In this report, we grouped patients using two different criteria, i.e., BCVA and ETDRS. We found that the distribution patterns of the three pathological mechanisms in the two classifications were significantly different. Only ischemia was correlated with disease severity as evaluated by the ETDRS criteria, and neurodegeneration were evenly distributed among ETDRS groups, which are in tandem with previous findings [30]. However, our results showed that neurodegeneration was an independent predictor of BCVA. Therefore, solely evaluating diabetic patients by vascular manifestation may lead to the risk that visual impairment could go undetected.

DR classification schemes have been extremely useful because they were designed in an era when the most essential issue was dealing with severe blinding retinopathy. The success of the classification schemes now brings us to the twenty-first century in which a consensus has been reached that treatment of DR should begin prior to the onset of vision-threatening stages [31, 32]. However, the conventional classification is based primarily on microvascular changes and does not incorporate recent findings of structural neuropathy in diabetes. Hence, the development of a new and comprehensive classification system of DR has been proposed [2]. Our results have provided fundamental evidence that monitoring neurodegeneration is of value for clinical endpoints and should be considered in the new and clinically useful classification scheme.

While both ischemia and neurodegeneration were independently correlated with decreased BCVA, they also interacted together to increase vision loss. Neurodegeneration may precede microvascular dysfunction in DR, and it may contribute to microvascular abnormalities [33]. The neurovascular unit may serve as the connection that links neurons and capillaries, and it may be the anatomical basis for the mutual influence of neurodegeneration and ischemia [34]. However, a more definitive understanding of the mutual influence of the two mechanisms needs to be clarified in future research.

It is a finding of interest that the OCT-measured subclinical edema, identified by retinal thickening compared to the normal control group, was not correlated with BCVA. Generally, diabetic macular edema is one of the main reasons that affect visual acuity of diabetic patients. However, subclinical edema was not related to BCVA, indicating timely control of edema in the subclinical course may prevent the occurrence of potential severe visual impairment. This hypothesis could be further verified in future studies.

We acknowledge several limitations of this study. First, the casual relationships between ischemia and neurodegeneration and the decrease of the BCVA were unclear due to the cross-sectional nature of the study and the limited number of subjects; thus, longitudinal studies of larger sample sizes are required to validate the findings. Second, we checked all OCT images and found no significant structural damage in the retinal layers. Nevertheless, some parameters such as ellipsoid zone disruption and disorganization of the retinal inner layers and the intercapillary area have been reported to be associated with loss of BCVA in DR. These potential factors, along with clinical characteristics such as axial length and HbA1c that could alter OCT and OCTA parameters and affect BCVA, should be considered in the future studies [35–37]. Third, although OCTA provided improved visualization of the microvasculature in different retinal layers compared to the traditional fundus images, especially in the early DR patients, it has limitations such as a small field of view in 3 × 3 mm, the influence of scan quality, and the inability to show the leakage. It is worth mentioning that fluorescein angiography is unsurpassed in the assessment of macular ischemia. Fluorescein angiography can often detect DR in patients that have no apparent DR on the basis of 7-field protocol using the ETDRS classification. Fourth, to minimize the impact of lens opacities on the decline of BCVA, we made a subjective assessment of lens status based upon expert analysis of slit-lamp biomicroscopy images, and only OCTA images with a signal strength index greater than 40 were chosen. The lack of lens status information that could have affected visual acuity may have confounded our findings.

## Conclusions

In conclusion, we demonstrated that subtle alterations in the microvasculature and neuroretina of DR eyes associated with decreased visual acuity can be detected quantitively by OCTA and OCT. Ischemia and neurodegeneration are critical factors that are related to the visual impairment and could exert a mutual influence over the natural course of the early stages of DR. These changes constitute prominent pathophysiological mechanisms in early DR, but they vary greatly among patients. A multimodal imaging protocol monitoring both microvascular alteration and neurodegenerative change is essential to identify the eyes at a higher risk for future vision loss, which will enhance the development of precision medicine in the management of DR.

## Supplementary Information

**Additional file 1: Fig. S1.** An example of significant artifact (SSI = 35). The yellow arrow shows the location of artifact (a). An example with SSI = 69 (b) was provided for comparison.

**Additional file 2: Table S1.** ROC analysis of microvascular parameters and retinal thickness of diabetic patients with normal and decreased BCVA.

## Data Availability

The datasets used and/or analyzed during the current study are available from the corresponding author on reasonable request.
