# Supplementary material for: Visual acuity is correlated with ischemia and neurodegeneration in patients with early stages of diabetic retinopathy
Source: Eye Vis (Lond). 2021 Oct 19;8:38. doi: 10.1186/s40662-021-00260-4 (PMC8527732; doi:10.1186/s40662-021-00260-4)

**Figure S1.** **An example of significant artifact (SSI=35).** The yellow arrow shows the location of artifact (a). An example with SSI=69 (b) was provided for comparison.


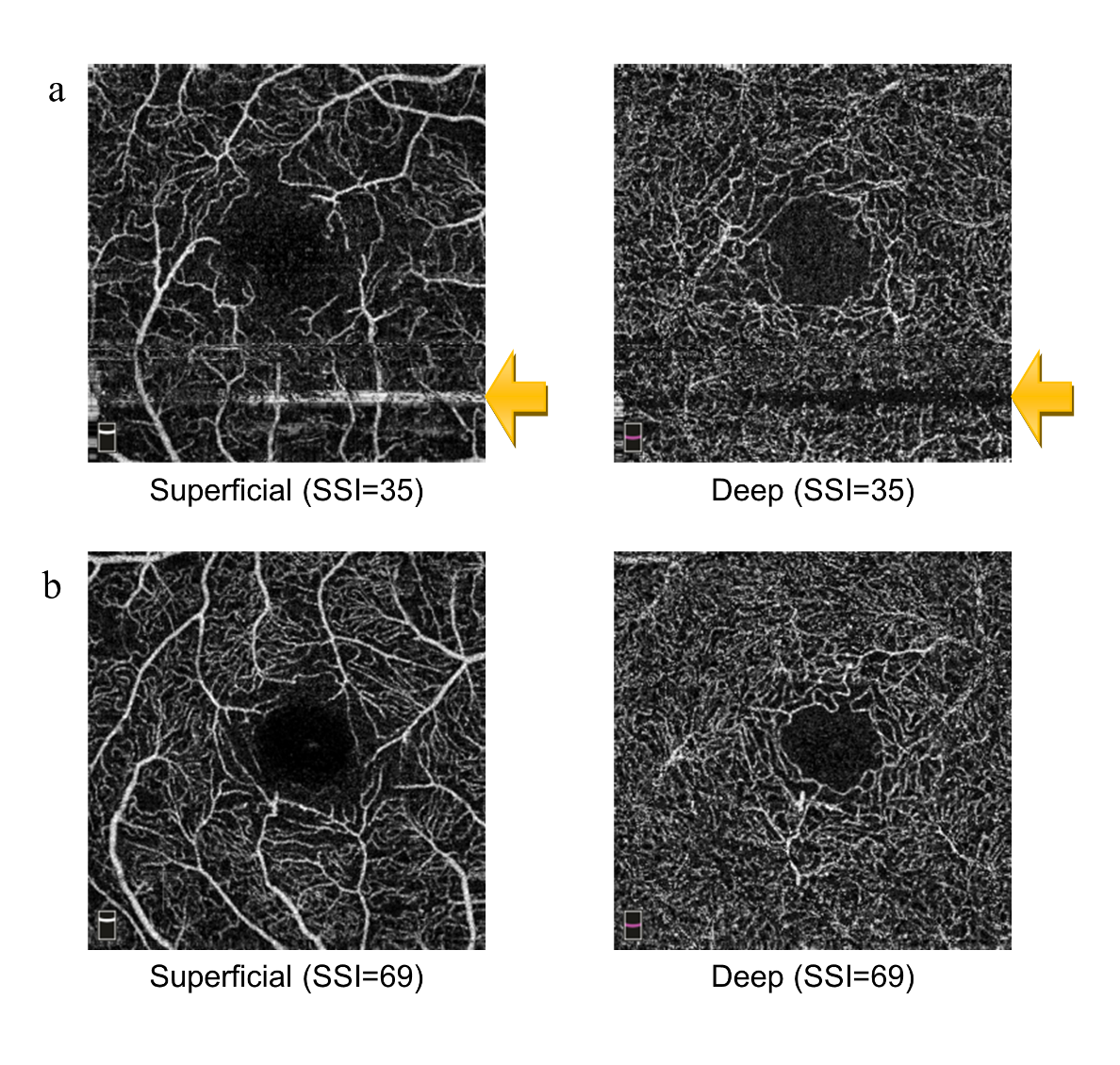

Supplement: Supplementary file 1 — Additional file 1: Fig. S1. An example of significant artifact (SSI = 35). The yellow arrow shows the location of artifact (a). An example with SSI = 69 (b) was provided for comparison. [file 40662_2021_260_MOESM1_ESM.docx]
