# Supplementary material for: Visual acuity is correlated with ischemia and neurodegeneration in patients with early stages of diabetic retinopathy
Source: Eye Vis (Lond). 2021 Oct 19;8:38. doi: 10.1186/s40662-021-00260-4 (PMC8527732; doi:10.1186/s40662-021-00260-4)
Supplement: Supplementary file 2 — Additional file 2: Table S1. ROC analysis of microvascular parameters and retinal thickness of diabetic patients with normal and decreased BCVA. [file 40662_2021_260_MOESM2_ESM.docx]

**Table S1. ROC analysis of microvascular parameters and retinal thickness of diabetic patients with normal and decreased BCVA.**

|  | **DR with normal BCVA** | | | |  | **DR with decreased VA** | | | |  |
| --- | --- | --- | --- | --- | --- | --- | --- | --- | --- | --- |
|  | **AUC** | **Cutoff** | **Sensitivity (%)** | **Specificity (%)** |  | **AUC** | **Cutoff** | **Sensitivity (%)** | **Specificity (%)** | |
| Microvascular parameters |  |  |  |  |  |  |  |  |  |  |
| VD SRCP | 0.637 | 0.055 | 60.61 | 69.70 |  | 0.640 | 0.053 | 61.36 | 68.18 |  |
| VD DRCP | 0.733 | 0.071 | 72.73 | 72.73 |  | 0.736 | 0.063 | 52.27 | 84.09 |  |
| FAZ area (mm²) | 0.601 | 0.270 | 85.61 | 39.39 |  | 0.680 | 0.335 | 77.27 | 55.68 |  |
| Retinal layer thickness |  |  |  |  |  |  |  |  |  |  |
| RNFL thickness (μm) | 0.622 | 28.393 | 62.12 | 60.61 |  | 0.580 | 29.273 | 61.36 | 57.95 |  |
| GCL-IPL thickness (μm) | 0.508 | 70.114 | 68.94 | 39.39 |  | 0.648 | 66.404 | 29.55 | 95.45 |  |
| INL thickness (μm) | 0.547 | 17.389 | 60.61 | 54.55 |  | 0.518 | 15.770 | 47.73 | 62.50 |  |
| OPL thickness (μm) | 0.560 | 10.993 | 42.42 | 78.79 |  | 0.526 | 10.047 | 50.00 | 60.23 |  |
| Total retinal thickness (μm) | 0.521 | 253.110 | 74.24 | 39.39 |  | 0.583 | 235.032 | 56.82 | 61.36 |  |

*ROC* receiver operating characteristic; *BCVA* best-corrected visual acuity; *DR* diabetic retinopathy; *AUC* area under the curve; *VD* vessel density; *SRCP* superficial retinal capillary plexus; *DRCP* deep retinal capillary plexus; *FAZ* foveal avascular zone; *RNFL* retinal nerve fiber layer; *GCL-IPL* ganglion cell layer plus inner plexiform layer; *INL* inner nuclear layer; *OPL* outer plexiform layer
